# MoRF-FUNCpred: Molecular Recognition Feature Function Prediction Based on Multi-Label Learning and Ensemble Learning

**DOI:** 10.3389/fphar.2022.856417

**Published:** 2022-03-08

**Authors:** Haozheng Li, Yihe Pang, Bin Liu, Liang Yu

**Affiliations:** ^1^ School of Computer Science and Technology, Xidian University, Xi’an, China; ^2^ School of Computer Science and Technology, Beijing Institute of Technology, Beijing, China; ^3^ Advanced Research Institute of Multidisciplinary Science, Beijing Institute of Technology, Beijing, China

**Keywords:** intrinsically disordered regions, molecular recognition features, multi-label learning, binary relevance, ensemble learning

## Abstract

Intrinsically disordered regions (IDRs) without stable structure are important for protein structures and functions. Some IDRs can be combined with molecular fragments to make itself completed the transition from disordered to ordered, which are called molecular recognition features (MoRFs). There are five main functions of MoRFs: molecular recognition assembler (MoR_assembler), molecular recognition chaperone (MoR_chaperone), molecular recognition display sites (MoR_display_sites), molecular recognition effector (MoR_effector), and molecular recognition scavenger (MoR_scavenger). Researches on functions of molecular recognition features are important for pharmaceutical and disease pathogenesis. However, the existing computational methods can only predict the MoRFs in proteins, failing to distinguish their different functions. In this paper, we treat MoRF function prediction as a multi-label learning task and solve it with the Binary Relevance (BR) strategy. Finally, we use Support Vector Machine (SVM), Logistic Regression (LR), Decision Tree (DT), and Random Forest (RF) as basic models to construct MoRF-FUNCpred through ensemble learning. Experimental results show that MoRF-FUNCpred performs well for MoRF function prediction. To the best knowledge of ours, MoRF-FUNCpred is the first predictor for predicting the functions of MoRFs. Availability and Implementation: The stand alone package of MoRF-FUNCpred can be accessed from https://github.com/LiangYu-Xidian/MoRF-FUNCpred.

## Introduction

Intrinsically disordered regions (IDRs) and intrinsically disordered proteins (IDPs) are sequence regions and proteins lack stable 3D structures (Deng et al., 2012; Deng et al., 2015). IDPs and IDRs are widely distributed in organisms. Research on IDPs and IDRs contributes to biomedicine and biology, such as drug discovery and protein structure prediction. Molecular recognition features (MoRFs) are regions that can make the IDR complete the transformation from disordered state to ordered state (Cheng et al., 2007). With the studies of MoRFs, these functional sites may play a role as druggable disease targets, and some drugs are discovered through these sites of action (Kumar et al., 2017; Li et al., 2020; Wang et al., 2020; Zhang et al., 2020; Lv et al., 2021a; Joshi et al., 2021; Shaker et al., 2021; Yan et al., 2021).

IDPs are widely found in eukaryotes, and traditional protein annotations do not consider disordered regions. Recent studies summarized the IDRs as seven functions: entropic chain, biological condensation, molecular recognition assembler (MoR_assembler), molecular recognition chaperone (MoR_chaperone), molecular recognition display sites (MoR_display_sites), molecular recognition effector (MoR_effector) and molecular recognition scavenger (MoR_scavenger), 1) entropic chain carries out some specific functions, these functions are generated by their conformational disorder, 2) molecular recognition assembler brings together multiple binding partners, promoting the formation of higher-order protein complexes, 3) molecular recognition scavenger can store and neutralizes some small ligands, 4) molecular recognition effector interacts with other proteins and to some extent influences their activity, 5) molecular recognition display sites is beneficial to deposition of post-translational modification, 6) molecular recognition chaperone can support RNA and protein to achieve functionally folded states, 7) biological condensation causes proteins to undergo transition from solution to condensed phase (Mohan et al., 2006; Van Der Lee et al., 2014; Hwang Fu et al., 2019; Canzhuang and Yonge, 2021; Gao et al., 2021; Luo et al., 2021; Qian D. et al., 2021; Qian L. et al., 2021; Sharma and Srivastava, 2021; Suresh et al., 2021; Wu et al., 2021). The MoR_assembler, MoR_chaperone, MoR_display_sites, MoR_effector, and MoR_scavenger of the 7 IDRs functions are MoRF functions (Mohan et al., 2006; Van Der Lee et al., 2014; Hwang Fu et al., 2019; Lv et al., 2019; Lv et al., 2020a; Kanathezath et al., 2021; Peng et al., 2021; Rives et al., 2021; Szklarczyk et al., 2021; Villegas-Morcillo et al., 2021).

Because of the potential biological significance of MoRFs, MoRF prediction methods have attracted increasing attention, such as OPAL (Sharma et al., 2018a), MoRFPred (Disfani et al., 2012), MoRFPred-Plus (Sharma et al., 2018b), MFPSSMpred (Fang et al., 2013), OPAL+ (Sharma et al., 2019), MoRFchibi (Malhis et al., 2016), and spot-MORF(Hanson et al., 2020). Although these methods can predict MoRFs in IDPs, they cannot distinguish their functions.

Some biological analyses are used in the existing methods of predicting MoRF functions; for example, through analysis of cellular viability by flow cytometry, a target’s function can be recognized (Johansson et al., 1998). Accurate prediction of the function of the MoRF region is conducive to understanding the mechanism of cancer and discovering targeted drugs. DisProt is a IDPs database. Disprot not only contains IDPs but also supports IDPs functional annotation (Piovesan et al., 2017). In our research, we found that these five MoRF functions are not mutually exclusive. Therefore, the prediction of MoRF function is a multi-label task. It is necessary to propose automatic discovery methods to expand the MoRF functional annotation.

In this study, we propose the first computational method for predicting the functions of MoRFs in IDPs called MoRF-FUNCpred. We introduce a method based on the residues of IDPs to predict the possibility that the residues have five functions of MoRFs. MoRF-FUNCpred uses an ensemble learning (Dietterich, 2000) model to predict the possibility of five functions of MoRFs. The individual classifiers are Support Vector Machine (SVM) (Vapnik and Vapnik, 1998), Logistic Regression (LR) (Cessie and Houwelingen, 1992), Decision Tree (DT) (Safavian and Landgrebe, 1991) and Random Forest (RF) (Breiman, 2001). The four models are integrated using a weighted averaging strategy, and the weights of the models are obtained through a genetic algorithm (Maulik and Bandyopadhyay, 2000).

The innovation of this work lies in the following: 1) we construct a dataset of inherently disordered proteins with MoRF functions annotation; 2) we take advantage of an ensemble model to integrate the different advantages of models; 3) we propose the first model, MoRF-FUNCpred, for predicting the functions of molecular recognition features in intrinsically disordered proteins.

## Materials and Methods

### Datasets

The data were extracted from the DisProt database, which is a database of IDPs and provides functional annotations of IDPs (Piovesan et al., 2017). The data can be downloaded from the site: https://disprot.org/api/search?release=2020_12&show_ambiguous=true&show_obsol (Hatos et al., 2020). In this version of the data, 1590 intrinsically disordered proteins were provided, and 596 proteins of them had functional annotations about disordered regions. The 7 functions of intrinsically disordered regions were divided into functions of MoRFs (MoR_assembler, MoR_chaperone, MoR_display_sites, MoR_effector and MoR_scavenger) and other functions (entropic chain and biological condensation).

After further screening of the 596 protein sequences obtained above, 3 proteins were deleted because of incorrect residue expression. Proteins with residues that only have both other functions and functions of MoRFs were deleted. To better construct the training set and testing set, some protein sequences with multi-MoRF functional residues were deleted, and finally, we obtained 565 sequences.

To reduce the similarity between the training set and the testing set, we ran BlastClust (Altschul et al., 1990) with length coverage >70% and identity threshold = 25% for the 565 sequences. Through this, we obtained 508 classes from 565 protein sequences. Next, we randomly divided the training set and the testing set according to the sequence number ratio of 1:1 based on the clustering result. Through this, we obtained a set containing 243 categories and another set containing 265 categories, including 283 pieces, and 282 pieces of sequences.

In this study, residue data were used as training data and testing data, and we selected residue data as follows: residues without 7 functions of IDRs were dropped, residues with both other functions and 5 functions of MoRFs were also dropped, residues with only 5 functions of MoRFs were selected as positive samples, and the other residues with only other functions were selected as negative samples. See Table 1 for the number of sequences with different functional residues and the number of different functional residues in the training set and testing set.

**TABLE 1:** Different functional residues in the protein sequence, training set, and testing set.

Types	Training set	Testing set	Sequences
Negative	6158	8108	167
MoR_assembler	8821	8537	160
MoR_chaperone	2006	1052	24
MoR_display_sites	1992	1503	58
MoR_effector	8431	7576	149
MoR_scavenger	1617	1500	20
MoR_assembler, MoR_ display_sites	301	294	9
MoR_assembler, MoR_effector	1134	434	17
MoR_display_sites, MoR_effector	1128	562	18
MoR_assembler, MoR_display_sites, MoR_effector	113	78	4

### Architecture of MoRF-FUNCpred

**FIGURE 1:**
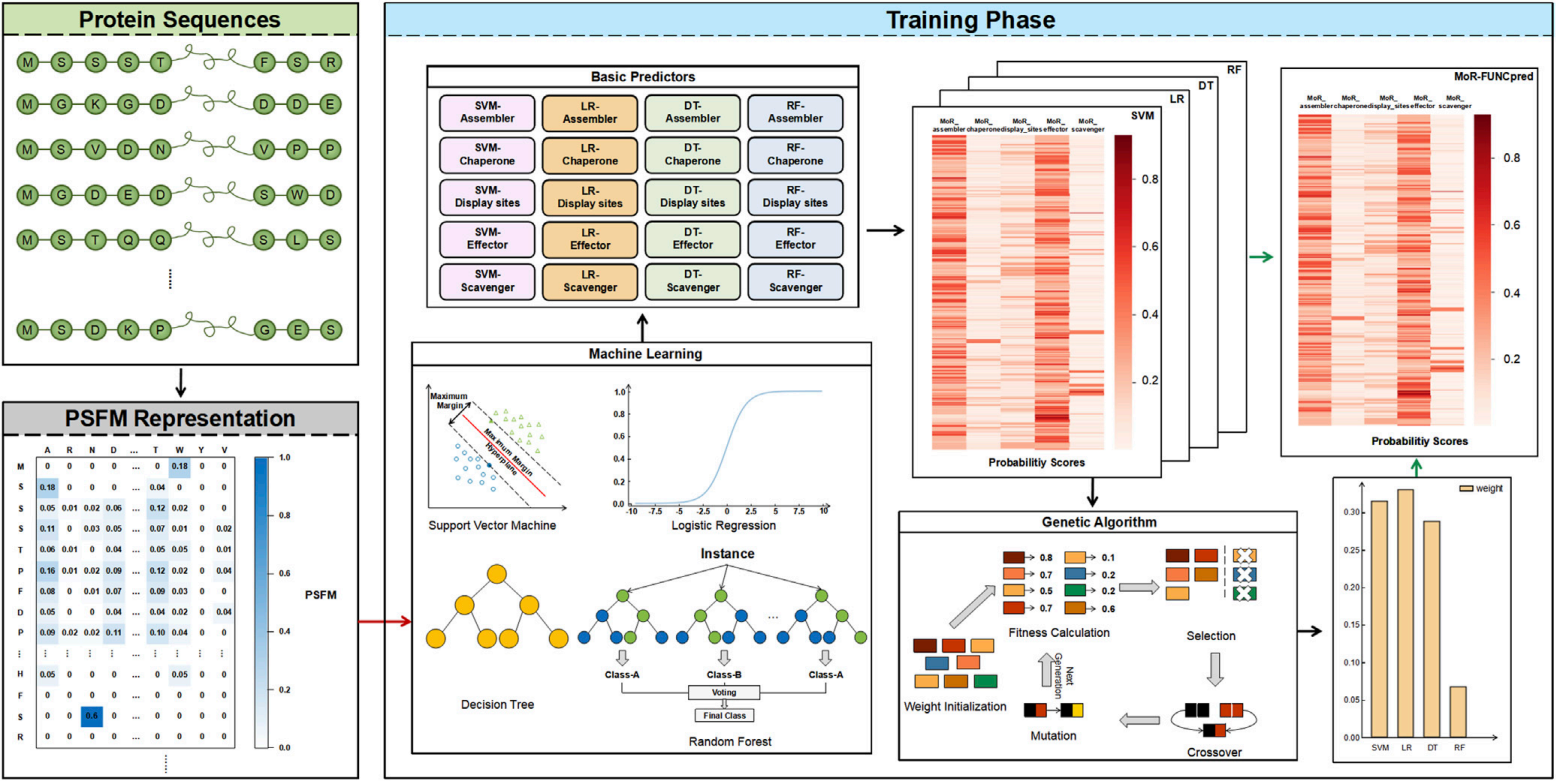
The network architecture of MoRF-FUNCpred. MoRF-FUNCpred uses PSFM to express the protein. MoRF-FUNCpred extracts features and labels of residues divided into training set and testing set. The SVM, LR, DT, and RF models are trained using the training set, and the four models are integrated to obtain better performance through ensemble learning. Obtain the weights of ensemble learning through the genetic algorithm.

The flowchart of MoRF-FUNCpred is shown in Figure 1, which includes protein sequences, PSFM representation and training phase.

#### PSFM Representation

In this study, protein evolutionary information was used as a protein sequence representation. The position specific frequency matrix (PSFM) is a kind of protein evolutionary information and indicates the frequency of 20 amino acids at the sequence corresponding position. PSFM has been used as a protein sequence representation in many studies (Wang et al., 2006; Liu et al., 2012; Zhu et al., 2019). In our paper, the PSFM was generated by using PSI-BLAST (Altschul et al., 1997) searching against the non-redundant database NRDB90 (Holm and Sander, 1998) with default parameters except that the iteration and e-value were as 10 and 0.001, respectively.

Protein sequence **P** of length *L* can be expressed as:

$$\begin{array}{c} P={\text{R}}_{1}{\text{R}}_{2}{\text{R}}_{3}\ldots {\text{R}}_{L} \end{array}$$ (1)

where R_*i*_ represents the amino acids of the protein sequence, and the subscript represents the *i*th residue in this protein.

The PSFM profile of protein **P** is a matrix, whose dimensions are *L* × 20:

$$\begin{array}{c} \mathrm{PSFM}=\left[\begin{array}{ccc} F_{1,1} & \cdots & F_{1,20}\\ \vdots & \ddots & \vdots \\ F_{L,1} & \cdots & F_{L,20} \end{array}\right]\; \end{array}$$ (2)

where 20 is the total number of standard amino acids. The element *F*_*i,j*_ is the probability of amino acid *j* occurring at position *i* of **P**.

#### Multi-Label Learning Strategy

The functions of MoRFs can be divided into five categories: MoR_assembler, MoR_chaperone, MoR_display_sites, MoR_effector and MoR_scavenger. According to the DisProt database, the MoRF functional regions overlap, leading to each residue carrying out multiple functions. Therefore, we treat MoRF functional prediction as a multi-label learning problem.

In this study, we wanted to make full use of positive samples. Therefore, the multi-label learning strategy “Binary Relevance” (BR) (Boutell et al., 2004) was employed. Under the “BR” strategy, the multi-label samples can be used as positive samples in each predictor of the corresponding label. We called this advantage “crossing training”.

In this paper, In order to explore the impact of different machine learning models on this task, four machine learning classifiers with the “BR” strategy were used to predict the probability of each MoRF function. Therefore, as Figure 1 shows, for each machine learning model, five classifiers are trained to predict different MoRF functions. We use the features of residues and the label of a certain MoRF function to train the classifier to obtain a classifier that can predict the corresponding function. Finally, 20 classifiers are trained in our model.

#### Ensemble Learning

Ensemble learning is used in many protein tasks and has good performance, such as recognition of multiple lysine PTM sites and the different types of these sites (Qiu et al., 2016a), recognition of phosphorylation sites in proteins (Qiu et al., 2016b) and recognition of protein folds (Liu et al., 2021). The ensemble model usually has better performance than individual predictors.

The flowchart of the ensemble strategy on different machine learning methods is given in the training phase of Figure 1.

#### Basic Classifiers

The general structure of ensemble learning is (i) generate a set of basic classifiers and (ii) select a combination strategy to ensemble basic classifiers. From the general structure of ensemble learning, we can find two common problems of ensemble learning. The first one is which basic classifiers to choose? The other is which combination strategies to select?

For the basic classifiers, we choose four common machine learning models: Support Vector Machine (SVM), Logistic Regression (LR), Decision Tree (DT) and Random Forest (RF). The four models are chosen because SVM can use the kernel trick to obtain nonlinear fitting ability, LR can solve the problem of linear fitting, DT usually has good performance in dealing with continuous features, and RF can balance errors when dealing with unbalanced datasets. To illustrate the complementarity of the four classifiers at the data level, we define the distance function between the classifiers (Liu et al., 2017):

$$\begin{array}{c} Distance\left(C(i),C(j)\right)=1-\frac{1}{2m}\overset{m}{\underset{k=1}{\sum }}d_{ik}\Delta d_{jk} \end{array}$$ (3)

where *m* represents the number of samples in the data, $d_{ik}$ represents the misclassification probability of classifier *C(i)* on the *k*th sample, and $d_{ik}\Delta d_{jk}$ can be calculated by (Liu et al., 2017):

$$\begin{array}{c} d_{ik}\text{Δ}d_{jk}=\{\begin{array}{c} d_{ik}+d_{jk},if\;C\left(i\right)\;and\;C\left(j\right)\;incorrectly\;predicts\;the\;kth\;sample\\ 0,\;otherwise \end{array}\; \end{array}$$ (4)

The value of Distance [*C*(*i*)*,C*(*j*)] ranges from 0 to 1, where 0 means that classifier *C*(*i*) and classifier *C*(*j*) are completely non-complementary, and 1 means that classifier *C*(*i*) and classifier *C*(*j*) are completely complementary (Liu et al., 2017). The value of Distance [*C*(*i*),*C*(*i*)] is between 0 and 1, Distance [*C*(*i*),*C*(*i*)] can reflect the predictive ability of classifier *C*(*i*), 1 means that classifier *C*(*i*) predicts all the data correctly, and 0 means that classifier *C*(*i*) predicts all the data incorrectly.

For the combination strategy, to make different models play the same role for each residue, the weighted averaging strategy was used to ensemble the 4 basic machine learning methods. The weighted averaging strategy can be represented as follows:

$$\begin{array}{c} MoRF-FUNCpred=W_{SVM}*SVM+W_{LR}*LR+W_{DT}*DT+W_{RF}*RF \end{array}$$ (5)

where $W_{SVM}$ , $W_{LR}$ , $W_{DT}$ and $W_{RF}$ represent the weight of each model in the ensemble model, the sum of the four values is 1, and *SVM*, *LR*, *DT*, and *RF* represent the 4 models that use the corresponding machine learning methods.

#### Genetic Algorithm

To obtain an optimal set of $W_{SVM}$ , $W_{LR}$ , $W_{DT}$ and $W_{RF}$ to maximize the *Macro_Accuracy* (see this metric in section *Performance Evaluation Strategy*) of MoRF-FUNCpred in the training set, we transform solving $W_{SVM}$ , $W_{LR}$ , $W_{DT}$ and $W_{RF}$ into a constrained optimization problem. Since the search space for this problem is large, the genetic algorithm is used to quickly obtain the optimal solution.

In our study, the *Macro_Accuracy* of the training set was used as the fitness, and the fitness was used to select outstanding individuals and eliminate individuals who were not adapted to the current environment. The characteristics of the better individuals will be passed on to the next generation. The genetic algorithm generates new individuals through crossover and mutation. In this way, the attributes that adapt to the environment are retained, and new attributes are introduced. After hundreds of circulations, the optimal weight can be obtained (Maulik and Bandyopadhyay, 2000).

The population size is set to 50, the constraint condition is $W_{SVM}+W_{LR}+W_{DT}+W_{RF}=1,\;0\leq W_{SVM}\leq 1,\;0\leq W_{LR}\leq 1,0\leq W_{DT}\leq 1,\;0\leq W_{RF},$ the mutation probability is 0.001, and the maximum number of iterations is 800.

### Performance Evaluation Strategy

In this paper, we use four metrics to measure the quality of a classifier: (i) accuracy of each function, (ii) overall metric *Macro_accuracy* to measure the performance of model, (iii) sensitivity (*sn*) to calculate the model’s performance of positive samples, (iv) specificity (*sp*) to represent the model’s quality of negative samples (Guo et al., 2020; Tao et al., 2020; Zhai et al., 2020; Wang et al., 2021; Yang et al., 2021).

The prediction of a residue by the model is a vector, and the dimension of the vector is 5. Each column is a fraction from 0 to 1 and represents the probability of residues with the MoR_assembler function, MoR_chaperone function, MoR_ display_sites function, MoR_effector function and MoR_scavenger function. The fraction can also be converted to a value of 0 or 1 by setting the threshold value to 0.5.

The accuracy of each function can be calculated by (Zhang and Zhou, 2013):

$$\begin{array}{c} Accuracy=\;\frac{TP+TN}{TP+FP+TN+FN} \end{array}$$ (6)

where *TP*, *TN*, *FP*, and *FN* is the number of “true” positive examples, the number of “true” negative examples, the number of “false” positive examples and the number of “false” negative examples, respectively. For multi-label task, macro accuracy (*Macro_Accuracy*) was selected to evaluate the overall performance of our model. *Macro_Accuracy* was calculated by Eq. 7:

$$\begin{array}{c} Macro\_Accuracy=\frac{\begin{array}{c} MoR\_assembler\_Accuracy+MoR\_chaperone\_Accuracy+\\ MoR\_display\_sites\_Accuracy+MoR\_effector\_Accuracy+\\ MoR\_scavenger\_Accuracy \end{array}}{N} \end{array}$$ (7)

where *MoR_assembler_Accuracy*, *MoR_chaperone_Accuracy*, *MoR_display_sites_Accuracy*, *MoR_effector_Accuracy*, and *MoR_scavenger_Accuracy* represent the accuracy of each function, and N represents the number of labels.

To calculate the prediction performance of the model for positive and negative samples of each function in the testing set, we calculated the sensitivity (*sn*) and specificity (*sp*) for each MoRF function (Jiang et al., 2013; Zhang and Zhou, 2013; Lv et al., 2020b; Tahir and Idris, 2020; Wan and Tan, 2020; Xie and Zhao, 2020; Lv et al., 2021b; Gao et al., 2021):

$$\begin{array}{c} sn=\frac{TP}{TP+FN}\; \end{array}$$ (8)

$$\begin{array}{c} sp=\frac{TN}{FP+TN} \end{array}$$ (9)

where *TP*, *TN*, *FP*, and *FN* is the number of “true” positive examples, the number of “true” negative examples, the number of “false” positive examples and the number of “false” negative examples, respectively.

We use *sn_MoR_assembler*, *sn_MoR_chaperone*, *sn_MoR_display_sites*, *sn_MoR_effector*, and *sn_MoR_scavenger* to represent the sensitivity for identifying the functions *MoR_assembler*, *MoR_chaperone*, *MoR_display_sites*, *MoR_effector* and *MoR_scavenger*, respectively. We use *sp_MoR_assembler*, *sp_MoR_chaperone*, *sp_MoR_display_sites*, *sp_MoR_effector* and *sp_MoR_scavenger* to represent the specificity for identifying the functions *MoR_assembler*, *MoR_chaperone*, *MoR_display_sites*, *MoR_effector* and *MoR_scavenger*, respectively.

## Results and Discussion

### Performance Comparison

We adjust the parameters of the four models in the training set based on the grid search strategy, and the parameters adopted to generate SVM were *C* = 16, *gamma* = 32, and *kernel* = rbf. The parameters adopted to generate LR were *penalty* = l2 and *c* = 0.03125. The parameters adopted to generate DT were *criterion* = gini and *splitter* = best. The parameters for generating RF were n_estimators = 80 and max_features = sqrt. See Table 2 for the value range of hyperparameters.

**TABLE 2:** Hyperparameter ranges for each model.

Model	Hyperparameters	Range
SVM	C	{2^−5^, 2^−4^, 2^−3^, 2^−2^, 2^−1^, 2^−0^, 2^1^, 2^2^, 2^3^, 2^4^, 2^5^}
	gamma	{2^−5^, 2^−4^, 2^−3^, 2^−2^, 2^−1^, 2^−0^, 2^1^, 2^2^, 2^3^, 2^4^, 2^5^}
	kernel	(liner, polynomial, rbf)
LR	penalty	{l1, l2}
	c	{2^−5^, 2^−4^, 2^−3^, 2^−2^, 2^−1^, 2^−0^, 2^1^, 2^2^, 2^3^, 2^4^, 2^5^}
DT	criterion	{gini, entropy}
	splitter	{best, random}
RF	n_estimators	{10, 20, 30, 40, 50, 60, 70, 80, 90, 100}
	max_features	{sqrt, log2}

We evaluate the overall metrics *Macro_Accuracy* and accuracy of each function (using *MoR_assembler_Accuracy*, *MoR_chaperone_Accuracy*, *MoR_display_sites_Accuracy*, *MoR_effector_Accuracy* and *MoR_scavenger_Accuracy* to represent the accuracy of different functions) of four basic models in the testing set. We can see the metrics of the four models in the testing set in Table 3.

**TABLE 3:** Different basic models in overall metric and accuracy of each function.

Metrics	SVM	LR	DT	RF
Macro_Accuracy	**0.828**	0.564	0.743	0.813
MoR_assembler_Accuracy	0.639	0.480	0.549	**0.640**
MoR_chaperone_Accuracy	**0.962**	0.620	0.877	0.930
MoR_display_sites_Accuracy	**0.918**	0.531	0.800	0.866
MoR_effector_Accuracy	0.684	0.580	0.598	**0.686**
MoR_scavenger_Accuracy	0.937	0.606	0.891	**0.944**

Bold values represent the best results for each metric.

From this table, we can find the following:

(i) A common phenomenon is that the prediction ability of different models in the MoR_assembler and MoR_effector functions is lower than that of the other three functions. The extremely important reason for this result is that for the MoR_assembler and MoR_effector functions, there are more positive samples in our dataset, and all models try to learn more information of positive samples. Although *Accuracy* is reduced, more positive samples are predicted correctly.
(ii) The difference between basic models is huge. SVM and RF have better performance than LR and DT not only in overall metric (*Macro_Accuracy*) but also in accuracy of each function. This is because different models try to predict different aspects; for example, some try to predict positive samples as much as possible, but others try to predict all negative samples.
(iii) The LR model in every metric is the worst of the four basic models, and in the MoR_assembler function prediction, the accuracy of the LR model is lower than 0.5. The huge gap between the SVM model and LR model probably shows that the PSFM feature is not strictly linearly separable in the task of MoRF function classification, and LR tries to predict more positive samples and causes low accuracy. However, LR model still have its’ advantage. To find more specific differences between each model, we use metrics *sn* and *sp* to see the extent to which positive and negative samples can be predicted for each function. Result are provided in Table 4.

**TABLE 4:** Comparison of the ensemble model and best metric in the single model.

Metrics	SVM	LR	DT	RF
sn_MoR_assembler	0.237	**0.493**	0.391	0.227
sp_MoR_assembler	0.824	0.474	0.622	**0.830**
sn_MoR_chaperone	0.003	**0.393**	0.099	0.029
sp_MoR_chaperone	**0.997**	0.629	0.906	0.963
sn_MoR_display_sites	0.000	**0.392**	0.287	0.213
sp_MoR_display_sites	**1.000**	0.544	0.846	0.925
sn_MoR_effector	0.150	**0.562**	0.291	0.109
sp_MoR_effector	0.902	0.587	0.725	**0.923**
sn_MoR_scavenger	0.047	**0.547**	0.109	0.022
sp_MoR_scavenger	0.984	0.609	0.932	**0.993**

Bold values represent the best results for each metric.

As we can see in Table 4, regardless of the proportion of positive and negative samples in the training data, the LR model’s result in the testing data changed less than that of the other models. In fact, the greatest advantage of LR is that its prediction ability is much better than that of the other three models in the positive samples. However, the LR model has poor performance in predicting negative samples. In contrast, SVM, DT, and RF are similar; these models have good results in negative samples, and in positive samples, the MoR_assembler and MoR_effector functions are better than the other models. Therefore, the differences between these models make it possible for us to ensemble learning.

*sn* of SVM, DT, RF model is low and *sp* of these models is high. When the positive samples of the MoRFs function are large, such as MoR_assembler and MoR_effector, *sn* will be higher than the other MoRF functions with less positive sample data, and *sp* will be lower than the other MoRF functions with less positive sample data.

### Complementarity of the Four Basic Classifiers

**FIGURE 2:**
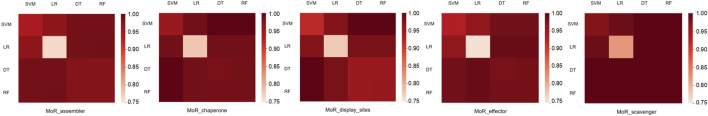
Distance between the two models in the training set under the five MoRF functions.

We calculate the distance between the two models in the training set under the five MoRF functions. The experimental results are shown in Figure 2. As seen from Figure 2,

(i) For each MoRF function, the distance between the same models is greater than 0.75, which shows that the four models themselves have good predictive capabilities.
(ii) The distance between different models of 5 MoRF functions is greater than 0.95, which shows that the two models are highly complementary.
(iii) DT and RF have similar distances to the four models. The main reason for this phenomenon is that the RF itself is a model formed by integrating many decision trees.

### Performance of Ensemble Model

We adopt the weighted average method in ensemble learning; that is, four weights for four models were set, and the sum of the weights was 1. The weight of each model represents the importance of each model. Through the genetic algorithm, we calculated that the weight of SVM was 0.31455477, the weight of LR was 0.32997175, the weight of DT was 0.28779645 and the weight of RF was 0.06767703.

The final ensemble learning results are shown in Table 5. We can see that in terms of overall indicators *Macro_Accuracy*, the ensemble learning results are better than the best results of a single model. However, we can also find that *MoR_chaperone_Accuracy* and *MoR_scavenger_Accuracy* are slightly worse than the best result in a single model; that is, because the ensemble model can obtain the best overall metric, it improves only some metrics. For example, it may enhance the accuracy of positive samples in some functions, and the price reduces the accuracy of negative samples in some functions. Because of the imbalanced dataset, improving the ability to predict positive samples cannot always improve the *sn* and *sp*.

**TABLE 5:** Comparison of the ensemble model and best metric in the single model.

Metrics	MoRF-FUNCpred	SVM	LR	DT	RF
Macro_Accuracy	**0.840**	0.828	0.564	0.743	0.813
MoR_assembler_Accuracy	**0.682**	0.639	0.480	0.549	0.640
MoR_chaperone_Accuracy	0.960	**0.962**	0.620	0.877	0.930
MoR_display_sites_Accuracy	0.910	**0.918**	0.531	0.800	0.866
MoR_effector_Accuracy	**0.703**	0.684	0.580	0.598	0.686
MoR_scavenger_Accuracy	**0.944**	0.937	0.606	0.891	**0.944**

Bold values represent the best results for each metric.

### Performance in Entire protein Sequence

**FIGURE 3:**
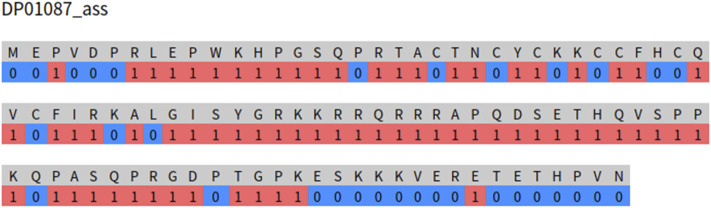
MoRF-FUNCpred prediction of the MoR_assembler function of DP01087.

MoRF-FUNCpred is trained using the PSFM features and the corresponding labels of the residues and screening the residues in the protein sequence. When providing an interface for other researchers to predict the MoRF functions of a protein, it is to input the entire protein sequence and predict the MoRF functions of the protein. MoRFs usually appear as sequence segments with 5–70 residues. Therefore, our MoRF function prediction should also appear as sequence segments with lengths of 5–70. To verify whether our prediction model also has this property, we randomly extract a sequence from the testing set and input it to the web server. As shown in Figure 3, we input the protein sequence signed DP01087. Three long sequence fragments were predicted as MoR_assembler functions, which is very similar to the MoR_assembler function of the real annotation results 1–101 in the disprot database, but there are still many discrete residue fragments predicted as MoR_assembler functions.

Therefore, although MoRF-FUNCpred inputs features and labels of residues, it still has the original sequence properties of MoRFs at the sequence level. From Figure 3, we can also find that there will still be several discrete residue prediction results that have the function of MoR_assembler. The reason for this phenomenon is mainly due to the input of our models and PSFM features.

The input of the model is features and labels of residues. Features of residues cannot completely reflect sequence properties. PSFM features are only used in MoRF-FUNCpred, and the ability of the PSFM features to capture sequence properties is limited, so MoRF-FUNCpred still has room for improvement.

## Conclusion

The existing methods for predicting the functions of MoRFs in IDP are mainly through analysis of cellular viability by flow cytometry. The problem with these methods is that the experimental period is long and the experimental cost is expensive. Predicting the functions of MoRFs by calculation methods can not only save time but also reduce experimental costs. We can use calculation methods to initially screen IDPs and further accurately measure the functions of MoRFs in cooperation with biological experiments.

In this study, the first MoRF function predictor is proposed called MoRF-FUNCpred, which predicts the functions of MoRFs regarding residues. MoRF-FUNCpred regards the residue MoRF function prediction task as a multi-label learning task. MoRF-FUNCpred uses PSFM features as the feature representation of residues and uses SVM, LR, DT, and RF combined with “BR” strategies to efficiently prepare for the completion of MoRF function prediction tasks. To utilize the complementarity between the models, the SVM, LR, DT, and RF are integrated through the weight method of ensemble learning, and the weight of each model is obtained through the genetic algorithm. Under the grid search for the best parameters for each model, in the single machine learning model (SVM, LR, DT, and RF), the overall metric *Macro_Accuracy* is greater than 0.5 for the prediction performance of MoRFs. Compared with single machine learning models, the ensemble model MoRF-FUNCpred shows better performance. In addition, although MoRF-FUNCpred is trained using residue data, the prediction results of MoRF-FUNCpred retain part of the sequence of MoRFs nature. At the same time, this paper constructs the first dataset on the function of MoRFs, which will provide help for further research on this task.

The main dilemma facing MoRF function prediction is that the existing IDPs containing MoRF functions are few, and it is difficult to complete the training tasks at the protein level. MoRF-FUNCpred mainly has the following problems. The use of a single feature of PSFM to represent residues may result in insufficient expression of residues. Using the “BR” strategy to complete the multi-label learning task may cause the model to ignore the correlation between the labels. In future work, we can explore the following aspects. 1) Use more complex features to represent residues, such as fusing multiple features to represent residues. This may enhance the expression ability of residues. 2) Using the high-order strategy in the multi-label learning problem transformation method, the model can learn the high-order correlation between the labels.

## Data Availability

The original contributions presented in the study are included in the article/Supplementary Material, further inquiries can be directed to the corresponding authors.

## References

[B1] Altschul S. F. Gish W. Miller W. Myers E. W. Lipman D. J. (1990). Basic Local Alignment Search Tool. J. Mol. Biol. 215, 403–410. 10.1016/S0022-2836(05)80360-2 2231712

[B2] Altschul S. F. Madden T. L. Schäffer A. A. Zhang J. Zhang Z. Miller W. (1997). Gapped BLAST and PSI-BLAST: a New Generation of Protein Database Search Programs. Nucleic Acids Res. 25, 3389–3402. 10.1093/nar/25.17.3389 9254694 PMC146917

[B3] Boutell M. R. Luo J. Shen X. Brown C. M. (2004). Learning Multi-Label Scene Classification. Pattern recognition. 37, 1757–1771. 10.1016/j.patcog.2004.03.009

[B4] Breiman L. (2001). Random Forests. Machine Learn. 45, 5–32. 10.1023/a:1010933404324

[B5] Canzhuang S. Yonge F. (2021). Identification of Disordered Regions of Intrinsically Disordered Proteins by Multi-Features Fusion. Curr. Bioinformatics 16, 1126–1132. 10.2174/1574893616666210308102552

[B6] Cessie S. L. Houwelingen J. C. V. (1992). Ridge Estimators in Logistic Regression. Appl. Stat. 41, 191–201. 10.2307/2347628

[B7] Cheng Y. Oldfield C. J. Meng J. Romero P. Uversky V. N. Dunker A. K. (2007). Mining alpha-helix-forming Molecular Recognition Features with Cross Species Sequence Alignments. Biochemistry 46, 13468–13477. 10.1021/bi7012273 17973494 PMC2570644

[B8] Deng X. Eickholt J. Cheng J. (2012). A Comprehensive Overview of Computational Protein Disorder Prediction Methods. Mol. Biosyst. 8, 114–121. 10.1039/c1mb05207a 21874190 PMC3633217

[B9] Deng X. Gumm J. Karki S. Eickholt J. Cheng J. (2015). An Overview of Practical Applications of Protein Disorder Prediction and Drive for Faster, More Accurate Predictions. Int. J. Mol. Sci. 16, 15384–15404. 10.3390/ijms160715384 26198229 PMC4519904

[B10] Dietterich T. G. (2000). “Ensemble Methods in Machine Learning,” in Proceedings of the International Workshop on Multiple Classifier Systems (Berlin, Heidelberg: Springer), 1–15.

[B11] Disfani F. M. Hsu W. L. Mizianty M. J. Oldfield C. J. Xue B. Dunker A. K. (2012). MoRFpred, a Computational Tool for Sequence-Based Prediction and Characterization of Short Disorder-To-Order Transitioning Binding Regions in Proteins. Bioinformatics. 28, i75–83. 10.1093/bioinformatics/bts209 22689782 PMC3371841

[B12] Fang C. Yamana H. Noguchi T. (2013). Sequence-based Prediction of Molecular Recognition Features in Disordered Proteins. J. Med. Bioeng. 2, 110–114. 10.12720/jomb.2.2.110-114

[B13] Gao J. Hu B. Chen L. (2021). A Path-Based Method for Identification of Protein Phenotypic Annotations. Curr. Bioinformatics 16, 1214–1222. 10.2174/1574893616666210531100035

[B14] Guo Z. Wang P. Liu Z. Zhao Y. (2020). Discrimination of Thermophilic Proteins and Non-thermophilic Proteins Using Feature Dimension Reduction. Front. Bioeng. Biotechnol. 8, 584807. 10.3389/fbioe.2020.584807 33195148 PMC7642589

[B15] Hanson J. Litfin T. Paliwal K. Zhou Y. (2020). Identifying Molecular Recognition Features in Intrinsically Disordered Regions of Proteins by Transfer Learning. Bioinformatics. 36, 1107–1113. 10.1093/bioinformatics/btz691 31504193

[B16] Hatos A. Hajdu-Soltész B. Monzon A. M. Palopoli N. Álvarez L. Aykac-Fas B. (2020). DisProt: Intrinsic Protein Disorder Annotation in 2020. Nucleic Acids Res. 48, D269–D276. 10.1093/nar/gkz975 31713636 PMC7145575

[B17] Holm L. Sander C. (1998). Removing Near-Neighbour Redundancy from Large Protein Sequence Collections. Bioinformatics. 14, 423–429. 10.1093/bioinformatics/14.5.423 9682055

[B18] Hwang Fu Y.-H. Chandrasekar S. Lee J. H. Shan S.-o. (2019). A Molecular Recognition Feature Mediates Ribosome-Induced SRP-Receptor Assembly during Protein Targeting. J. Cel Biol. 218, 3307–3319. 10.1083/jcb.201901001 PMC6781444 31537711

[B19] Jiang Q. Wang G. Jin S. Li Y. Wang Y. (2013). Predicting Human microRNA-Disease Associations Based on Support Vector Machine. Int. J. Data Min Bioinform. 8, 282–293. 10.1504/ijdmb.2013.056078 24417022

[B20] Johansson J. Gudmundsson G. H. Rottenberg M. E. Berndt K. D. Agerberth B. (1998). Conformation-dependent Antibacterial Activity of the Naturally Occurring Human Peptide LL-37. J. Biol. Chem. 273, 3718–3724. 10.1074/jbc.273.6.3718 9452503

[B21] Joshi P. Vedhanayagam M. Ramesh R. (2021). An Ensembled SVM Based Approach for Predicting Adverse Drug Reactions. Curr. Bioinformatics 16, 422–432. 10.2174/1574893615999200707141420

[B22] Kanathezath A. Chembra V. Padingare Variyath S. K. Nair G. G. (2021). Identification of Biomarkers and Functional Modules from Genomic Data in Stage-wise Breast Cancer. Curr. Bioinformatics 16, 722–733. 10.2174/1574893615999200922123104

[B23] Kumar D. Sharma N. Giri R. (2017). Therapeutic Interventions of Cancers Using Intrinsically Disordered Proteins as Drug Targets: C-Myc as Model System. Cancer Inform. 16, 1176935117699408. 10.1177/1176935117699408 28469390 PMC5392011

[B24] Li Z. Zhang T. Lei H. Wei L. Liu Y. Shi Y. (2020). Research on Gastric Cancer's Drug-Resistant Gene Regulatory Network Model. Curr. Bioinformatics 15, 225–234. 10.2174/1574893614666190722102557

[B25] Liu B. Wang S. Long R. Chou K. C. (2017). iRSpot-EL: Identify Recombination Spots with an Ensemble Learning Approach. Bioinformatics. 33, 35–41. 10.1093/bioinformatics/btw539 27531102

[B26] Liu B. Wang X. Chen Q. Dong Q. Lan X. (2012). Using Amino Acid Physicochemical Distance Transformation for Fast Protein Remote Homology Detection. PLOS ONE 7 (9), e46633. 10.1371/journal.pone.0046633 23029559 PMC3460876

[B27] Liu Y. Han K. Zhu Y.-H. Zhang Y. Shen L.-C. Song J. (2021). Improving Protein Fold Recognition Using Triplet Network and Ensemble Deep Learning. Brief. Bioinform. 22, bbab248. 10.1093/bib/bbab248 34226918 PMC8768454

[B28] Luo Y. Wang X. Li L. Wang Q. Hu Y. He C. (2021). Bioinformatics Analysis Reveals Centromere Protein K Can Serve as Potential Prognostic Biomarker and Therapeutic Target for Non-small Cell Lung Cancer. Curr. Bioinformatics 16, 106–119. 10.2174/1574893615999200728100730

[B29] Lv Z. Ao C. Zou Q. (2019). Protein Function Prediction: from Traditional Classifier to Deep Learning. Proteomics. 19, e1900119. 10.1002/pmic.201900119 31187588

[B30] Lv Z. Cui F. Zou Q. Zhang L. Xu L. (2021a). Anticancer Peptides Prediction with Deep Representation Learning Features. Brief. Bioinform. 22, bbab008. 10.1093/bib/bbab008 33529337

[B31] Lv Z. Ding H. Wang L. Zou Q. (2021b). A Convolutional Neural Network Using Dinucleotide One-Hot Encoder for Identifying DNA N6-Methyladenine Sites in the rice Genome. Neurocomputing. 422, 214–221. 10.1016/j.neucom.2020.09.056

[B32] Lv Z. Wang P. Zou Q. Jiang Q. (2020a). Identification of Sub-golgi Protein Localization by Use of Deep Representation Learning Features. Bioinformatics. 36, 5600–5609. 10.1093/bioinformatics/btaa1074 PMC8023683 33367627

[B33] Lv Z. Zhang J. Ding H. Zou Q. (2020b). RF-PseU: a Random forest Predictor for RNA Pseudouridine Sites. Front. Bioeng. Biotechnol. 8, 134. 10.3389/fbioe.2020.00134 32175316 PMC7054385

[B34] Malhis N. Jacobson M. Gsponer J. (2016). MoRFchibi SYSTEM: Software Tools for the Identification of MoRFs in Protein Sequences. Nucleic Acids Res. 44, W488–W493. 10.1093/nar/gkw409 27174932 PMC4987941

[B35] Maulik U. Bandyopadhyay S. (2000). Genetic Algorithm-Based Clustering Technique. Pattern recognition. 33, 1455–1465. 10.1016/s0031-3203(99)00137-5

[B36] Mohan A. Oldfield C. J. Radivojac P. Vacic V. Cortese M. S. Dunker A. K. (2006). Analysis of Molecular Recognition Features (MoRFs). J. Mol. Biol. 362, 1043–1059. 10.1016/j.jmb.2006.07.087 16935303

[B37] Peng J. Xue H. Wei Z. Tuncali I. Hao J. Shang X. (2021). Integrating Multi-Network Topology for Gene Function Prediction Using Deep Neural Networks. Brief Bioinform 22, 2096–2105. 10.1093/bib/bbaa036 32249297

[B38] Piovesan D. Tabaro F. Mičetić I. Necci M. Quaglia F. Oldfield C. J. (2017). DisProt 7.0: a Major Update of the Database of Disordered Proteins. Nucleic Acids Res. 45, D219–D227. 10.1093/nar/gkw1056 27899601 PMC5210544

[B39] Qian D. Li Q. Zhu Y. Li D. (2021). Comprehensive Analysis of Key Proteins Involved in Radioresistance of Prostate Cancer by Integrating Protein-Protein Interaction Networks. Curr. Bioinformatics 16, 139–145. 10.2174/1574893615999200605143510

[B40] Qian L. Jiang Y. Xuan Y. Y. Yuan C. SiQiao T. (2021). PsePSSM-based Prediction for the Protein-ATP Binding Sites. Curr. Bioinformatics 16, 576–582. 10.2174/1574893615999200918183543

[B41] Qiu W. R. Sun B. Q. Xiao X. Xu Z. C. Chou K. C. (2016a). iPTM-mLys: Identifying Multiple Lysine PTM Sites and Their Different Types. Bioinformatics. 32, 3116–3123. 10.1093/bioinformatics/btw380 27334473

[B42] Qiu W. R. Xiao X. Xu Z. C. Chou K. C. (2016b). iPhos-PseEn: Identifying Phosphorylation Sites in Proteins by Fusing Different Pseudo Components into an Ensemble Classifier. Oncotarget. 7, 51270–51283. 10.18632/oncotarget.9987 27323404 PMC5239474

[B43] Rives A. Meier J. Sercu T. Goyal S. Lin Z. Liu J. (2021). Biological Structure and Function Emerge from Scaling Unsupervised Learning to 250 Million Protein Sequences. Proc. Natl. Acad. Sci. U S A. 118, e2016239118. 10.1073/pnas.2016239118 33876751 PMC8053943

[B44] Safavian S. R. Landgrebe D. (1991). A Survey of Decision Tree Classifier Methodology. IEEE Trans. Syst. Man. Cybern. 21, 660–674. 10.1109/21.97458

[B45] Shaker B. Tran K. M. Jung C. Na D. (2021). Introduction of Advanced Methods for Structure-Based Drug Discovery. Curr. Bioinformatics 16, 351–363. 10.2174/1574893615999200703113200

[B46] Sharma A. K. Srivastava R. (2021). Protein Secondary Structure Prediction Using Character Bi-gram Embedding and Bi-LSTM. Curr. Bioinformatics 16, 333–338. 10.2174/1574893615999200601122840

[B47] Sharma R. Bayarjargal M. Tsunoda T. Patil A. Sharma A. (2018a). MoRFPred-plus: Computational Identification of MoRFs in Protein Sequences Using Physicochemical Properties and HMM Profiles. J. Theor. Biol. 437, 9–16. 10.1016/j.jtbi.2017.10.015 29042212

[B48] Sharma R. Raicar G. Tsunoda T. Patil A. Sharma A. (2018b). OPAL: Prediction of MoRF Regions in Intrinsically Disordered Protein Sequences. Bioinformatics. 34, 1850–1858. 10.1093/bioinformatics/bty032 29360926

[B49] Sharma R. Sharma A. Raicar G. Tsunoda T. Patil A. (2019). OPAL+: Length-specific MoRF Prediction in Intrinsically Disordered Protein Sequences. Proteomics. 19, e1800058. 10.1002/pmic.201800058 30324701

[B50] Suresh N. T. Ravindran V. E. Krishnakumar U. (2021). A Computational Framework to Identify Cross Association between Complex Disorders by Protein-Protein Interaction Network Analysis. Curr. Bioinformatics 16, 433–445. 10.2174/1574893615999200724145434

[B51] Szklarczyk D. Gable A. L. Nastou K. C. Lyon D. Kirsch R. Pyysalo S. (2021). The STRING Database in 2021: Customizable Protein-Protein Networks, and Functional Characterization of User-Uploaded Gene/measurement Sets. Nucleic Acids Res. 49, D605–D612. 10.1093/nar/gkaa1074 33237311 PMC7779004

[B52] Tahir M. Idris A. (2020). MD-LBP: an Efficient Computational Model for Protein Subcellular Localization from HeLa Cell Lines Using SVM. Curr. Bioinformatics 15, 204–211. 10.2174/1574893614666190723120716

[B53] Tao Z. Li Y. Teng Z. Zhao Y. (2020). A Method for Identifying Vesicle Transport Proteins Based on LibSVM and MRMD. Comput. Math. Methods Med. 2020, 8926750. 10.1155/2020/8926750 33133228 PMC7591939

[B54] Van Der Lee R. Buljan M. Lang B. Weatheritt R. J. Daughdrill G. W. Dunker A. K. (2014). Classification of Intrinsically Disordered Regions and Proteins. Chem. Rev. 114, 6589–6631. 10.1021/cr400525m 24773235 PMC4095912

[B55] Vapnik V. N. Vapnik V. (1998). Statistical Learning Theory, 1. New York: Wiley.

[B56] Villegas-Morcillo A. Makrodimitris S. van Ham R. C. H. J. Gomez A. M. Sanchez V. Reinders M. J. T. (2021). Unsupervised Protein Embeddings Outperform Hand-Crafted Sequence and Structure Features at Predicting Molecular Function. Bioinformatics. 37, 162–170. 10.1093/bioinformatics/btaa701 32797179 PMC8055213

[B57] Wan X. Tan X. (2020). A Simple Protein Evolutionary Classification Method Based on the Mutual Relations between Protein Sequences. Curr. Bioinformatics 15, 1113–1129. 10.2174/1574893615666200305090055

[B58] Wang B. Chen P. Huang D. S. Li J. J. Lok T. M. Lyu M. R. (2006). Predicting Protein Interaction Sites from Residue Spatial Sequence Profile and Evolution Rate. FEBS Lett. 580, 380–384. 10.1016/j.febslet.2005.11.081 16376878

[B59] Wang J. Shi Y. Wang X. Chang H. (2020). A Drug Target Interaction Prediction Based on LINE-RF Learning. Curr. Bioinformatics 15, 750–757. 10.2174/1574893615666191227092453

[B60] Wang X. Yang Y. Liu J. Wang G. (2021). The Stacking Strategy-Based Hybrid Framework for Identifying Non-coding RNAs. Brief. Bioinform. 22, bbab023. 10.1093/bib/bbab023 33693454

[B61] Wu C. Lin B. Shi K. Zhang Q. Gao R. Yu Z. (2021). PEPRF: Identification of Essential Proteins by Integrating Topological Features of PPI Network and Sequence-Based Features via Random Forest. Curr. Bioinformatics 16, 1161–1168. 10.2174/1574893616666210617162258

[B62] Xie X. Zhao Y. (2020). A 2D Non-degeneracy Graphical Representation of Protein Sequence and its Applications. Curr. Bioinformatics 15, 758–766. 10.2174/1574893615666200106114337

[B63] Yan N. Lv Z. Hong W. Xu X. (2021). Feature Representation and Learning Methods with Applications in Protein Secondary Structure. Front. Bioeng. Biotechnol. 9, 748722. 10.3389/fbioe.2021.748722 34568304 PMC8458893

[B64] Yang H. Luo Y. Ren X. Wu M. He X. Peng B. (2021). Risk Prediction of Diabetes: Big Data Mining with Fusion of Multifarious Physical Examination Indicators. Inf. Fusion. 75, 140–149. 10.1016/j.inffus.2021.02.015

[B65] Zhai Y. Chen Y. Teng Z. Zhao Y. (2020). Identifying Antioxidant Proteins by Using Amino Acid Composition and Protein-Protein Interactions. Front Cel Dev Biol. 8, 591487. 10.3389/fcell.2020.591487 PMC7658297 33195258

[B66] Zhang M.-L. Zhou Z.-H. (2013). A Review on Multi-Label Learning Algorithms. IEEE Trans. Knowledge Data Eng. 26, 1819–1837. 10.1109/tkde.2013.39

[B67] Zhang S. Su M. Sun Z. Lu H. Zhang Y. (2020). The Signature of Pharmaceutical Sensitivity Based on ctDNA Mutation in Eleven Cancers. Exp. Biol. Med. (Maywood) 245, 720–732. 10.1177/1535370220906518 32050795 PMC7221488

[B68] Zhu X.-J. Feng C.-Q. Lai H.-Y. Chen W. Hao L. (2019). Predicting Protein Structural Classes for Low-Similarity Sequences by Evaluating Different Features. Knowledge-Based Syst. 163, 787–793. 10.1016/j.knosys.2018.10.007

